# Expression of LINC00847 in Peripheral Blood Mononuclear Cells of Children with Asthma and Its Prediction between Asthma Exacerbation and Remission

**DOI:** 10.1155/2022/5678257

**Published:** 2022-03-20

**Authors:** Jiaying Hu, Zhike Wang, Suzhen Han, Kai Chen

**Affiliations:** Department of Pediatrics, Ningbo Yinzhou No. 2 Hospital, Ningbo City, Zhejiang Province 315192, China

## Abstract

**Objective:**

Asthma is defined as a heterogeneous disease that is usually characterized by chronic airway inflammation. Long noncoding RNAs play important roles in various biological processes including inflammation. To know more about the relationships between lncRNAs and asthma, we sought to the role of LINC00847 in peripheral blood mononuclear cells (PBMCs) of children with asthma exacerbation or asthma remission.

**Methods:**

Microarray analysis was performed on GSE143192 and GSE165934 datasets to screen differentially expressed lncRNAs (DElncRNAs) in human PBMCs between asthma patients and normal controls. LINC00847 was selected from DElncRNAs in human PBMCs between asthma patients and normal controls for further investigation. The expression levels of LINC00847 were quantified in PBMCs collected from 54 children with asthma exacerbation, 54 children with asthma remission, and 54 healthy children by real-time qPCR. The forced expiratory volume in the first second in percent predicted values (FEV1%), ratio of forced expiratory volume in 1 second to forced vital capacity (FEV1/FVC), and peak expiratory flow rate (PEF%) were tested for evaluation of lung function. The concentration of immunoglobulin E (IgE) and eosinophil count was examined. The serum levels of interleukin-4 (IL-4), interferon-*γ* (IFN-*γ*), and IL-17A were determined by the ELISA method.

**Results:**

The expression level of LINC00847 in PBMCs of asthma exacerbation children was remarkably higher than that in PBMCs of asthma remission children and healthy children (*p* < 0.001); the expression level of LINC00847 in PBMCs of asthma remission children was notably higher than that in PBMCs of healthy children (*p* < 0.001). Pearson correlation analysis revealed that the expression levels of LINC00847 in PBMCs of asthma children were negatively correlated with FEV1% (*r* = −0.489), FEV1/FVC (*r* = −0.436), PEF% (*r* = −0.626), and IFN-*γ* level (*r* = −0.614) of asthma children, but positively correlated with IgE concentration (*r* = 0.680), eosinophil count (*r* = 0.780), IL-4 (*r* = 0.524), and IL-17A (*r* = 0.622) levels. When LINC00847 expression was used to distinguish asthma exacerbation from asthma remission, a 0.871 AUC (95% CI: 0.805–0.936) was yielded with sensitivity of 79.63% and specificity of 77.78%.

**Conclusion:**

The study demonstrates that increased LINC00847 expression may be associated with the development and progression of asthma, possibly serving as a novel biomarker for predicting asthma exacerbation from asthma remission.

## 1. Introduction

Asthma is a chronic respiratory disease with dyspnea caused by airway hyperresponsiveness and inflammation. It is characterized by paroxysmal and reversible wheezing, chest tightness, shortness of breath, and cough, which affects people of all ages [1]. Asthma is a common disease among children. The incidence rate and morbidity of asthma are closely related to the prolongation of time. In 2016, 9.6% of children aged 5–11 and 10.5% of children aged 12–17 were reported to suffer from asthma [2]. Asthma in preschool children is prone to worsen, but the injury is relatively limited, while older children and adolescents have more diseases mainly caused by the injuries [3]. Asthma is usually found in early childhood. It was reported that asthmatics with symptoms such as wheezing in the first 6 years after birth is as high as 50%, and about 40% of children still have wheezing in late childhood [4]. In fact, wheezing and cough are not the main ways to identify asthma. Children without asthma, especially for children aged 0–2 years, also have these symptoms due to respiratory tract infection [5]. Various factors, including the disease history, family history, respiratory symptoms, spirometry, and serum tests such as immunoglobulin E (IgE) level and blood eosinophils, should be used as the diagnostic criteria of asthma [6]. Standard treatment has been successfully applied to most asthmatic children, but a considerable number of children have other serious diseases and are resistant to this treatment [7]. A study of over 10 years for development of asthma in children demonstrated that 0.5% of children had severe asthma in all 10-year-olds, and overall prevalence of severe asthma was 4.5% [8]. According to the severity and clinical presentation, asthma can be classified as asthma exacerbation or asthma remission. Asthma exacerbation is characterized by coughing, exercise intolerance, and recurrent episodes of increased respiratory effort at rest, representing the exacerbation of the condition, alternated with periods of remission of the clinical signs [9]. Children with severe asthma are more at risk of drug-related side effects, life-threatening deterioration, and impaired quality of life [10, 11]. Asthma is an immune-related disease. Compared with adults with asthma, children with severe asthma have significantly higher eosinophil number, allergen sensitization, and IgE level [12]. In addition, a previous study proved that elevated level of interleukin-4 (IL-4) and interleukin-17 (IL-17) and decreased level of interferon-*γ* (IFN-*γ*) were associated with pathogenesis of allergic asthma [13].

The prevalence of asthma is associated with genetic and environmental factors [14, 15]. Many studies have reported that microRNAs (miRNAs) and long noncoding RNAs (lncRNAs) were involved in a variety of biological processes [16, 17]. LncRNAs, as miRNAs sponges, inhibit the function of targeted miRNAs via binding to specific miRNAs, so as to regulate the expression of downstream target genes [18]. The function of lncRNAs has been widely revealed in numerous diseases such as coronary artery disease [19], breast cancer [20], and neurological disease [21]. Some research studies on lung cancer, chronic obstructive pulmonary disease, asthma, tuberculosis, and interstitial lung disease demonstrated that lncRNAs have made progress in the pathogenesis of respiratory diseases [22–24].

In recent years, overexpression of a newly identified lncRNAs LINC00847 has been associated with the occurrence of cancer [25, 26]. However, the function and expression of LINC00847 on the development of asthma has been rarely reported. This study contributed to prove LINC00847 is a highly expressed lncRNA in children with asthma in clinical and explore the correlation between LINC00847 and lung function, as well as immunity.

## 2. Materials and Methods

### 2.1. Microarray Dataset Preparation and Differential Expression Analysis

We download GSE143192 (submission date: January 6, 2020; last update date: July 31, 2021) and GSE165934 (submission date: February 01, 2021) from the Gene Expression Omnibus (GEO, https://www.ncbi.nlm.nih.gov/gds). The GSE143192 was generated on the GPL22120 platform, containing peripheral blood mononuclear cells (PBMCs) from 4 asthma patients and 4 healthy controls. The GSE165934 was generated on the GPL23126 platform, including PBMCs from 10 patients with bronchial asthma and 9 healthy controls. Differentially expressed lncRNAs (DElncRNAs) in human PBMCs between asthma patients and normal controls were sorted by analyzing raw data of GSE143192 and GSE165934 using the affy and limma package from the R/Bioconductor software with log |fold change (FC)| ≥ 1.2 and an adjusted *p* value less than 0.05 as cutoff. Venn intersect function was used to characterize overlapping lncRNAs between GSE143192 and GSE165934 datasets after differential expression analysis.

### 2.2. Study Subjects

This study included 54 children with asthma exacerbation and 54 children with asthma remission who were admitted into our hospital June 2019 to June 2021, and all of them fulfilled two inclusion criteria: diagnosis of asthma exacerbation or asthma remission according to the guidelines for diagnosis, prevention, and treatment of bronchial asthma in children (2016) and age ranging from 5 to 14 years. Those who had respiratory tract infection and systemic infection, used immunosuppressive drugs in a recent month, were complicated with allergic rhinitis or eczema, had a family history of asthma, or had undergone surgery were excluded from the study. Among 54 children with asthma exacerbation, there were 28 male children and 26 female children, aged 7.63 ± 2.29 years. Among 54 children with asthma remission, there were 30 male children and 24 female children, aged 7.39 ± 2.11 years. Normal controls were those who were confirmed as healthy children by physical examinations in our hospital June 2019 to June 2021, consisting of 30 male children and 24 female children, aged 7.50 ± 2.16 years. The children in the three groups were age and gender-matched. Written informed consent was provided by the patient or their legal guardian before data collection. The study was approved by the ethics committee of our hospital.

### 2.3. Lung Function Test

The lung function test were performed by the MS-IOS Digital instrument (Erich Jager AG, Würzburg, Germany), including the forced expiratory volume in the first second in percent predicted values (FEV1%), ratio of forced expiratory volume in 1 second to forced vital capacity (FEV1/FVC), and peak expiratory flow rate (PEF%).

### 2.4. Blood Sample Collection

Fasting venous blood (4 mL) was collected from asthma children and healthy children in morning. One part of each blood sample was placed into the tube and underwent 1200 r/m centrifugation at 4°C for 15 min. The serum was isolated and stored at −80°C. Another part of each blood sample was placed into the EDTA-contained tube. PBMCs were isolated using Ficoll density gradient centrifugation and stored at −80°C.

### 2.5. Enzyme-Linked Immunosorbent Assay

The concentration of IgE was detected by the IMMAGE800 automatic specific protein analyzer (Beckman Coulter, USA). The eosinophil count was examined by the LH500 hematology analyzer (Beckman Coulter). The serum levels of IL-4, IFN-*γ*, and IL-17A were determined by the ELISA method using commercially available kits (Abcam, UK).

### 2.6. RNA Extraction and Real-Time qPCR (RT-qPCR)

Total RNA was extracted from PBMCs by a mean of TRIzol (Invitrogen, USA) and qualified by the NanoDrop 2000c spectrophotometer (Thermo-Fisher Scientific, Waltham, MA, USA). A PrimeScript RT Reagent kit (Takara, Dalian, China) was used to generate cDNA from extracted total RNA. Synthesized cDNA was amplified on the ABI7500 Real-Time PCR Instrument (Applied Biosystems, USA) using the SYBR^®^ Premix Ex Taq™ II (Tli RNaseH Plus) kit (Takara). The reaction system consisted of 2 *μ*L cDNA, 10 *μ*L Real-Time Master Mix, and forward and reverse primers each for 1 *μ*L, supplemented with RNAase-free DEPC. The qPCR reaction was carried out according to the following cycling conditions: 95°C for 10 min followed by 40 cycles at 95°C for 15 s, 60°C for 1 min, and 72°C for 30 s. Primer sequences were as follows: LINC00847-forward (5′-AACGCTGCCTCTGTGGAAGTCTC-3′) and reverse (5′-CGCTCTGCTCTCCCGCCATC-3′); GAPDH-forward (5′-TATAAATTGAGCCCGCAGCC-3′) and reverse (5′-TACGACCAAATCCGTTGACTC-3′). The expression level of LINC00847 was presented by relative fold change to GAPDH based on the 2^−∆∆Ct^ method.

### 2.7. Statistical Analysis

Data in this study consisted of enumeration data and measurement data. Enumeration data were calculated by frequency analysis, and two groups were compared by the chi-square test. Measurement data were presented as mean ± standard deviation and analyzed by either the unpaired test or ANOVA/post hoc. Relationships between expression of LINC00847, FEV1%, FEV1/FVC, PEF%, IgE, eosinophil count, IL-4, IFN-*γ*, and IL-17A were evaluated by Pearson correlation analysis. The area under the receiver operating characteristic (ROC) curve (AUC) was used as a performance measure of expression of LINC00847 in predicting asthma exacerbation from asthma remission. Statistically significant differences were reflected by a value of *p* < 0.05.

## 3. Results

### 3.1. Identification of DElncRNAs in PBMCs between Asthma and Normal Children

**Figure 1:**
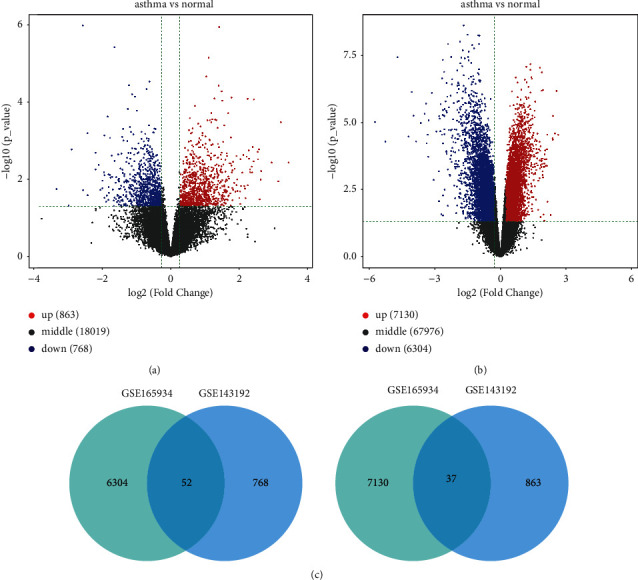
Identification of DElncRNAs in PBMCs between asthma and normal children. (a) The volcano plot showing a total of 1631 DElncRNAs in PBMCs between asthma and normal children after differential expression analysis on the GSE143192 dataset. (b) The volcano plot showing a total of 13434 differentially expressed genes in PBMCs between asthma and normal children after differential expression analysis on the GSE165934 dataset. (c) Venn plots showing overlapping 52 downregulated DElncRNAs and 37 upregulated DElncRNAs in PBMCs between asthma and normal children by analyzing the GSE143192 and GSE165934 datasets.

After differential expression analysis on the GSE143192 dataset, a total of 1631 lncRNAs fulfilled log |FC| ≥ 1.2 and adjusted *p* < 0.05 and stood out as DElncRNAs in PBMCs between asthma and normal children, including 863 upregulated DElncRNAs and 768 downregulated DElncRNAs (Figure 1(a)). After differential expression analysis on the GSE165934 dataset, a total of 13434 genes fulfilled log |FC| ≥ 1.2 and adjusted *p* < 0.05 and stood out as differentially expressed genes in PBMCs between asthma and normal children, including 7130 upregulated DElncRNAs and 6304 downregulated DElncRNAs (Figure 1(b)). Venn intersect function was used to characterize overlapping lncRNAs between 1631 DElncRNAs and 13434 differentially expressed genes. It was found that common 52 downregulated DElncRNAs and 37 upregulated DElncRNAs stood out (Figure 1(c)). Therefore, we selected LINC00847 for further investigation.

### 3.2. Increased Expression Levels of LINC00847 in PBMCs of Asthma Children

**Figure 2:**
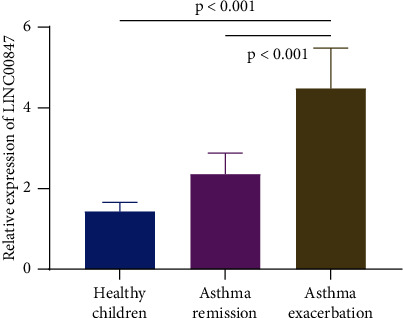
The expression level of LINC00847 in PBMCs of asthma exacerbation children, asthma remission children, and healthy children.

The expression level of LINC00847 in PBMCs of asthma exacerbation children, asthma remission children, and healthy children was determined by RT-qPCR. The expression levels of LINC00847 were 4.48 ± 0.99, 2.36 ± 0.51, and 1.43 ± 0.23 in PBMCs of asthma exacerbation children, asthma remission children, and healthy children, respectively. It was found that the expression level of LINC00847 in PBMCs of asthma exacerbation children was remarkably higher than that in PBMCs of asthma remission children and healthy children (*p* < 0.001); the expression level of LINC00847 in PBMCs of asthma remission children was notably higher than that in PBMCs of healthy children (*p* < 0.001, Figure 2).

### 3.3. The Expression Level of LINC00847 Was Associated with Lung Function of Asthma Children

**Figure 3:**
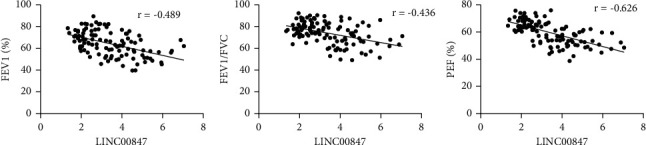
Pearson correlation analysis of the expression level of LINC00847 with lung function of asthma children.

FEV1%, FEV1/FVC, and PEF% were evaluated to reflect the lung function of asthma exacerbation children, asthma remission children, and healthy children. As given in Table 1, the FEV1%, FEV1/FVC, and PEF% of asthma exacerbation children were lower than those of asthma remission children and healthy children (*p* < 0.001); the FEV1%, FEV1/FVC, and PEF% of asthma remission children were lower than those of healthy children (*p* < 0.001). It was revealed by Pearson correlation analysis that the expression levels of LINC00847 in PBMCs of asthma children were negatively correlated with the FEV1%, FEV1/FVC, and PEF% of asthma children (Figure 3).

### 3.4. The Expression Level of LINC00847 Was Associated with Immune Function of Asthma Children

**Figure 4:**
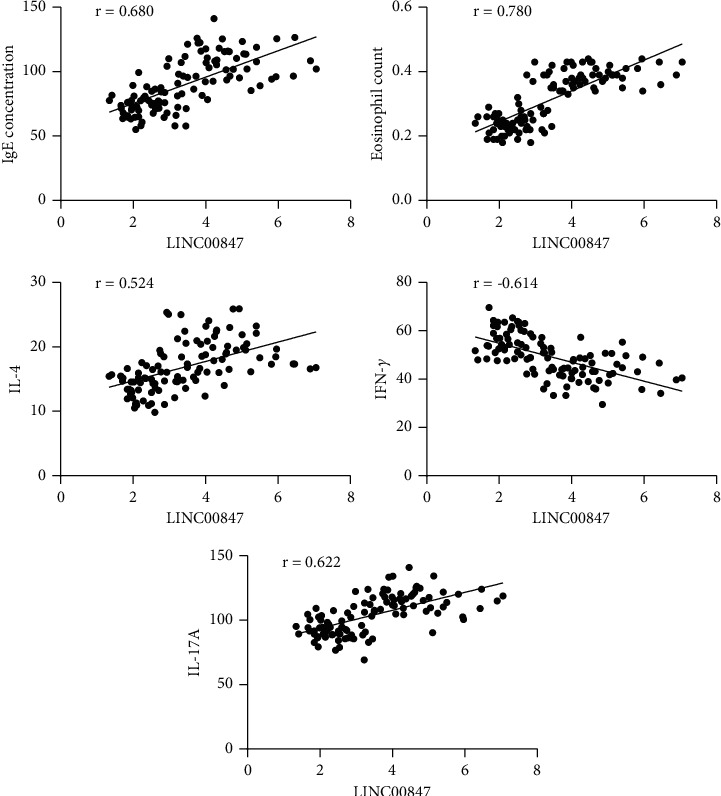
Pearson correlation analysis of the expression level of LINC00847 with IgE concentration, eosinophil count, IL-4, IFN-*γ*, and IL-17A of asthma children.

The immune function of asthma children and healthy children was evaluated by detecting IgE concentration, eosinophil count, IL-4, and IL-17A. The asthma exacerbation children exhibited higher IgE concentration, eosinophil count, IL-4, and IL-17A levels than asthma remission children and healthy children (*p* < 0.001); the asthma remission children had higher IgE concentration, eosinophil count, IL-4, and IL-17A levels than healthy children (*p* < 0.001, Table 2). A lower level of IFN-*γ* was noted in asthma exacerbation children than asthma remission children and healthy children (*p* < 0.001); the asthma remission children showed a lower level of IFN-*γ* than healthy children (*p* < 0.001). After Pearson correlation analysis, it was revealed that the expression levels of LINC00847 in PBMCs of asthma children were positively correlated with IgE concentration, eosinophil count, IL-4, and IL-17A levels, but negatively correlated with the IFN-*γ* level of asthma children (Figure 4).

### 3.5. Prediction of Asthma Exacerbation from Asthma Remission by LINC00847 Expression

**Figure 5:**
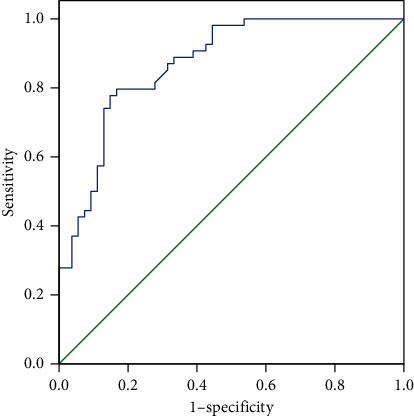
The AUC used as a performance measure of LINC00847 expression in predicting asthma exacerbation from asthma remission.

The AUC was used as a performance measure of LINC00847 expression in predicting asthma exacerbation from asthma remission. As shown in Figure 5, when LINC00847 expression was used to distinguish asthma exacerbation from asthma remission, a 0.871 AUC (95% CI: 0.805–0.936) was yielded with sensitivity of 79.63% and specificity of 77.78%.

## 4. Discussion

Asthma is a complex heterogeneous disease with high incidence rate in children. A study on global prevalence and severity of asthma symptoms in children indicated that the occurrence of severe asthma in Asia-Pacific, Northern and Eastern Europe, and North America was 3.8% and 11.3%, respectively [27]. Low-to-medium dose of inhaled corticosteroid has been successfully used to treat up to 95% of asthmatic children; however, such therapy has been ineffective in nearly 45% of children with asthma sometimes, and they experienced at least one episode of poorly controlled asthma [28, 29]. Severe asthma not only imposes a serious burden on medical treatment but also has adverse consequences on children, including drug-related side effects, reduced quality of life, and life-threatening deterioration caused by poor control [30, 31]. Asthma is caused by the interaction of a series of genetic and environmental factors. Numerous evidences proved that lncRNAs were involved in immune response, inflammation, and progression of immune-related diseases such as asthma [16, 32].

In this study, we collected the data of differential expression of lncRNAs in human peripheral blood mononuclear cells (PBMCs) between asthma patients and healthy groups using gene database platform and identified the differential expression of a novel lncRNAs LINC00847 in asthmatic patients. In order to further analyze the correlation between LINC00847 and asthma in children, children with asthma exacerbation, children with asthma remission, and healthy children were selected as the subjects of this study. LINC00847 is a newly identified lncRNA which located on 5q35.3, showing tissue-specific expression patterns [33]. Increasing studies have confirmed that changes in lncRNA LINC00847 expression affect the progression of cancer and might be used as a potential apoptosis biomarker in renal cell carcinoma [25], hepatocellular carcinoma [34], and nonsmall cell lung cancer [26]. This study determined LINC00847 expression in PBMCs of all the studied children by RT-qPCR. The results manifested that higher expression of LINC00847 was found in children with asthma exacerbation than asthma remission children and healthy children. Expression of LINC00847 increased with the severity of asthma. These findings were similar to another research by Wen et al. who observed that upregulated expression of three particular lncRNAs, including LINC00847, MIR22HG, and LNC_00027, was associated with microcystin-LR-mediated liver damage [35].

Asthma is a chronic respiratory disease. The measurement of lung function is very important for evaluating lung injury in patients with asthma. FEV1% measurement is a common method to evaluate asthma and chronic obstructive pulmonary disease [36]. Expiratory flow is mainly produced by abdominal muscle activity, which minimizes the risk of infection by expelling foreign bodies from the lungs [37]. The present study found that the asthma exacerbation children had much lower value of FEV1%, FEV1/FVC, and PEF% compared to asthma remission children and healthy children. The level of these measurements was highest in healthy children. This result was indirectly confirmed by a study on airway disorders, demonstrating nebulization with saline solution contributed to improve level of FEV1, FVC, and PEF in children with mild respiratory ailments [38]. Inadequate and excessive immune response is the basis of a variety of diseases, such as severe infection, metastatic malignancy, and asthma [39]. It was observed that, in this study, higher IgE concentration, eosinophil count, IL-4, and IL-17A levels were revealed in the asthma exacerbation children than in the remaining children; what is more, these levels increased with the severity of asthma. Compared to the remaining children, the asthma exacerbation children showed lower level of IFN-*γ*. Highest level of IFN-*γ* was indicated in the healthy children. Hattori et al. pointed out sustained expression of IFN-*γ*-induced favorable immune changes and improved atopic dermatitis in mice [40]. IL plays an indispensable role in the pathogenesis of inflammation. Gandhi et al. explored that the secretion of proinflammatory IL-17 cytokines and anti-inflammatory IL-4 cytokines, as biomarkers of airway inflammation potentially regulate asthma symptoms [41]. A report on severe eosinophilic asthma indicated that increasing eosinophil counts rather than elevated serum IgE concentrations increased the exacerbation risk for patients [42]. The outcomes were a little different from our analysis due to different types of asthma and patients' age. Mummadi et al. revealed that variability in IgE concentrations in patients with severe asthma affected therapeutic dose; thus, repeating serum IgE determinations were necessary for specific patients [43].

This study performed Pearson correlation analysis to find out the correlation between LINC00847 and lung function and immune function. The analysis indicated value of FEV1%, FEV1/FVC, and PEF% was negatively correlated with expression of LINC00847. In addition, LINC00847 expression was positively correlated with serum IgE concentration, eosinophil count, IL-4, and IL-17A level, but negatively correlated with the IFN-*γ* level. This study confirmed the role of LINC00847 expression in predicting asthma remission to asthma deterioration through AUC. These findings showed that LINC00847 was involved in the occurrence of asthma, which affected the regulation of immune response, airway inflammation, and other pathological processes related to asthma.

In summary, lncRNAs LINC00847 participates in the occurrence and development of childhood asthma by mediating intercellular communication and is closely related to immunity, inflammatory response, and apoptosis. It is a new molecule to be observed in the treatment of childhood asthma. In the near future, the interaction between lncRNAs LINC00847 and miRNAs in asthma can be further analyzed to determine the specific mechanism of targeted gene on childhood asthma.

## Figures and Tables

**Table 1:** The FEV1%, FEV1/FVC, and PEF% of asthma exacerbation children, asthma remission children, and healthy children.

Item	Healthy children (*n* = 54)	Asthma remission (*n* = 54)	Asthma exacerbation (*n* = 54)	*P*_1_	*P*_2_
FEV1%	93.13 ± 10.16	70.82 ± 9.15	57.69 ± 9.21	<0.001	<0.001
FEV1/FVC	93.63 ± 8.58	79.38 ± 6.70	68.34 ± 10.08	<0.001	<0.001
PEF%	86.38 ± 3.24	65.62 ± 5.58	53.44 ± 5.94	<0.001	<0.001

FEV1%, forced expiratory volume in the first second in percent predicted values; FEV1/FVC, ratio of forced expiratory volume in 1 second to forced vital capacity; PEF%, peak expiratory flow rate; *P*_1_, asthma remission compared to healthy children; *P*_2_, asthma exacerbation compared to asthma remission.

**Table 2:** The IgE concentration, eosinophil count, IL-4, IFN-*γ*, and IL-17A of asthma exacerbation children, asthma remission children, and healthy children.

Item	Healthy children (*n* = 54)	Asthma remission (*n* = 54)	Asthma exacerbation (*n* = 54)	*P*_1_	*P*_2_
IgE (U/mL)	23.27 ± 5.01	74.09 ± 9.45	105.65 ± 14.16	<0.001	<0.001
Eosinophil (×10^9^/L)	0.14 ± 0.03	0.24 ± 0.03	0.39 ± 0.03	<0.001	<0.001
IL-4 (ng/L)	7.99 ± 2.18	14.25 ± 2.16	19.49 ± 3.22	<0.001	<0.001
IFN-*γ* (ng/L)	69.44 ± 6.92	55.59 ± 5.58	42.96 ± 5.64	<0.001	<0.001
IL-17A (ng/L)	52.85 ± 13.92	91.67 ± 7.80	115.68 ± 9.34	<0.001	<0.001

*P*_1_, asthma remission compared to healthy children; *P*_2_, asthma exacerbation compared to asthma remission.

## Data Availability

The data used to support the findings of this study are included within the article.
